# Inflammatory vaginitis in women on long-term rituximab treatment for autoimmune disorders

**DOI:** 10.1186/s12905-021-01423-0

**Published:** 2021-08-05

**Authors:** Laura Yockey, Sarah Dowst, Reza Zonozi, Noah Huizenga, Patrick Murphy, Karen Laliberte, Jillian Rosenthal, John L. Niles, Caroline M. Mitchell

**Affiliations:** 1 Department of Medicine, Massachusetts General Hospital, Boston, MA USA; 2 Division of Nephrology, Vasculitis and Glomerulonephritis Clinic, Massachusetts General Hospital, Boston, MA USA; 3 Department of Obstetrics and Gynecology, Vincent Center for Reproductive Biology, Massachusetts General Hospital, 55 Fruit St, Boston, MA 02114 USA

## Abstract

**Background:**

Consequences of long-term B cell depletion with rituximab are not well understood. We describe inflammatory vaginitis as a potential side effect of long-term rituximab treatment, distinct from previously described vulvovaginal pyoderma gangrenosum.

**Methods:**

We performed a retrospective analysis of women treated with rituximab for more than 1 year to determine the prevalence and clinical characteristics of vaginitis cases. We conducted a case–control analysis with up to 3 controls for each vaginitis case.

**Results:**

We identified sixteen inflammatory vaginitis cases. Women with vaginitis were age 23–68 (median 42), primarily being treated for ANCA-associated vasculitis (11/16; 69%). Most reported copious vaginal discharge (100%) and pain with sex (75%). All women with return of circulating B-cells to > 10 cells/mL had complete (5/9) or significant (4/9) improvement in symptoms. In case–control analysis there was no significant difference in length of B-cell depletion, immune parameters, creatinine levels, and history of neutropenia.

**Conclusion:**

Inflammatory vaginitis is a potential side effect of prolonged continuous B cell depletion with rituximab. More studies are needed to characterize the incidence and etiology of vaginitis among women on long term rituximab therapy and establish a causal relationship.

## Key points

- Inflammatory vaginitis is a potential complication of long-term continuous B cell depletion with rituximab.
- Women being treated with rituximab should be screened for symptoms such as vaginal discharge and vaginal pain.

## Background

B cell depletion with rituximab, a monoclonal anti-CD20 antibody, induces remission for several antibody-mediated autoimmune diseases, including anti-neutrophil cytoplasmic autoantibody (ANCA)-associated vasculitis (AAV) and rheumatoid arthritis [1, 2]. Due to the relapsing nature of many of these diseases, long-term B cell depletion with repeated doses has been utilized successfully to maintain remission [3]. However, with increasing use of B cell depletion, there is a growing recognition of treatment-related complications. Hypogammaglombulinemia, opportunitistic infections, viral reactivation, and late-onset neutropenia (LON) are well described adverse events, but newer complications continue to emerge [4].

Mucocutaneous complications of the vagina and vulva have been reported in women on rituximab. The main clinical entity described to date has been vulvovaginal pyoderma gangrenosum [5–8]. Clinically, it presents with painful vulvar and/or perianal ulcerations with vaginal discharge and irritation. Biopsy demonstrates neutrophilic dermatoses, and patients are treated with intravenous immune globulin (IVIG), glucocorticoids, and/or discontinuation of rituximab. However, these descriptions are limited to a few case reports, and remain poorly understood.

Herein, we describe a novel mucocutanous complication of rituximab—inflammatory vaginitis—distrinct from the previously reported pyoderma grangrenosum. We define inflammatory vaginitis as bothersome vaginal symptoms with no identifiable cause, usually with high numbers of white blood cells (WBC) in vaginal fluid. We compared women with inflammatory vaginitis to matched controls from a cohort of 454 women undergoing rituximab treatment for autoimmune disease to characterize clinical features associated with the vaginitis. This study highlights a debilitating complication of B cell depletion not previously described.

## Methods

Cases were identified from a database of patients treated with rituximab for autoimmune disease between November 8, 2002 and April 29, 2020 at the Vasculitis and Glomerulonephritis Center (VGC) of Massachusetts General Hospital, a tertiary-care referral and treatment center. Patients were included in the analysis if they were female, 25–90 years old, and had received at least four doses of rituximab, which typically corresponds to 1 year of B cell depletion based on a schedule of 2 doses administered 2–4 weeks apart followed by one dose every 4–6 months [4]. Cases of inflammatory vaginitis were identified as the unexplained presence of bothersome vaginal symptoms (discharge, pain, or irritation). To determine if patients had another identifiable etiology of vaginal complaints, medical records were reviewed for results of vaginal wet mount, vaginal pH, testing for other infections, and biopsies. Controls were female patients without a history of documented vaginal complaints identified from the same database over the same time period. Each case was matched with up to 3 randomly selected controls based on year of birth (within 5 years), underlying autoimmune disease, and number of doses of rituximab. The study was approved by the Mass General Brigham Human Research Committee.

Covariates extracted from the electronic medical record included the duration of continuous B cell depletion (CD19 + CD20 + cell count, < 5 cells/uL)[4], number of prior infections, number of late-onset neutropenia (LON) episodes, cumulative cyclophosphamide dose, serum immunoglobulins (IgG, IgM, IgA), white blood cell count (WBC), T cell count, and serum creatinine levels at the time of vaginitis diagnosis for cases and at the corresponding time in matched controls.

Continuous variables are reported as median (interquartile range [IQR]) or mean (standard deviation), as appropriate. Categorical variables are reported as number (%). Differences in clinical parameters between cases and controls were assessed using Chi-squared test, Student’s t-test, or Mann–Whitney U test, where appropriate. All comparisons are two-tailed, with p < 0.05 considered significant. Analyses were carried out using STATA version 15 (College Station, Texas).

## Results

We identified 454 women treated with rituximab for autoimmune disease, of whom 279 (61%) had ANCA-associated vasculitis, 32 (7%) had membranous nephropathy, 21 (4.6%) had minimal change disease or idiopathic focal segmental glomerulosclerosis, 18 (4%) had lupus nephritis, and 104 (23%) had another diagnosis or multiple diagnoses. Out of 454 patients, 16 patients (3.5%) developed inflammatory vaginitis. Ten additional patients endorsed vaginal complaints attributable to another identifiable etiology, including bacterial vaginosis, vulvovaginal candidiasis, Reiter’s syndrome, vaginal prolapse, vulvar dermatitis, and vulvovaginal atrophy, and thus were excluded from the analysis. A significantly higher proportion of patients with age less than 50 years (8/96) were identified as having inflammatory vaginitis compared to patients 50 and older (8/358) (8% vs 2%; *p* = 0.004).

All patients reported vaginal discharge. Other symptoms included pain or dyspareunia (12/16; 75%), burning (7/16; 44%), and irritation (6/16; 38%). For 12 women, one or more full gynecological exams were available for review. On examination of the vaginal fluid at the time of most severe findings, 9/12 (75%) had a pH > 5 and 11/12 (92%) had more white blood cells (WBCs) than epithelial cells present. Many women (8/12, 67%) had parabasal cells present on wet mount analysis. Additionally, 9/12 (75%) had no lactobacilli present, indicating non-ideal altered vaginal flora. Of patients tested for sexually transmitted infections all were negative: 5 for *N. gonorrhoeae* and *C. trachomatis*, 5 for syphilis, and 12 for trichomonas. Of those tested for yeast infections using either molecular assays or culture, 4/12 (33%) had at least one positive culture but also had additional negative tests despite ongoing symptoms. Two patients had biopsies which showed “spongiotic dermatitis with mixed neutrophilic, lymphocytic, and eosinophilic inflammation and neutrophilic exocytosis” and “lichenoid dermatitis” of the vagina and vulva with negative immunohistochemsitry for herpes simplex and varicella zoster virus. One patient was hospitalized for IV analgesics due to the severity of the vaginal pain.

Patients were diagnosed with vaginitis 11–119 months after starting rituximab infusions (median 44) and 31–256 months (median 86 months) after their initial diagnosis. The majority of patients (15/16, 94%) received cyclophosphamide as part of their induction protocol. A total of 9 patients were taking or had previously taken one or more additional immunosuppressive drugs prior to developing vaginitis. Other immunosuppressive drugs included mycophenolate (3/16, 19%), azathioprine (5/16, 31%), and methotrexate (3/16, 19%). One patient also had a history of receiving abatacept and adalimumab.

**Fig. 1:**
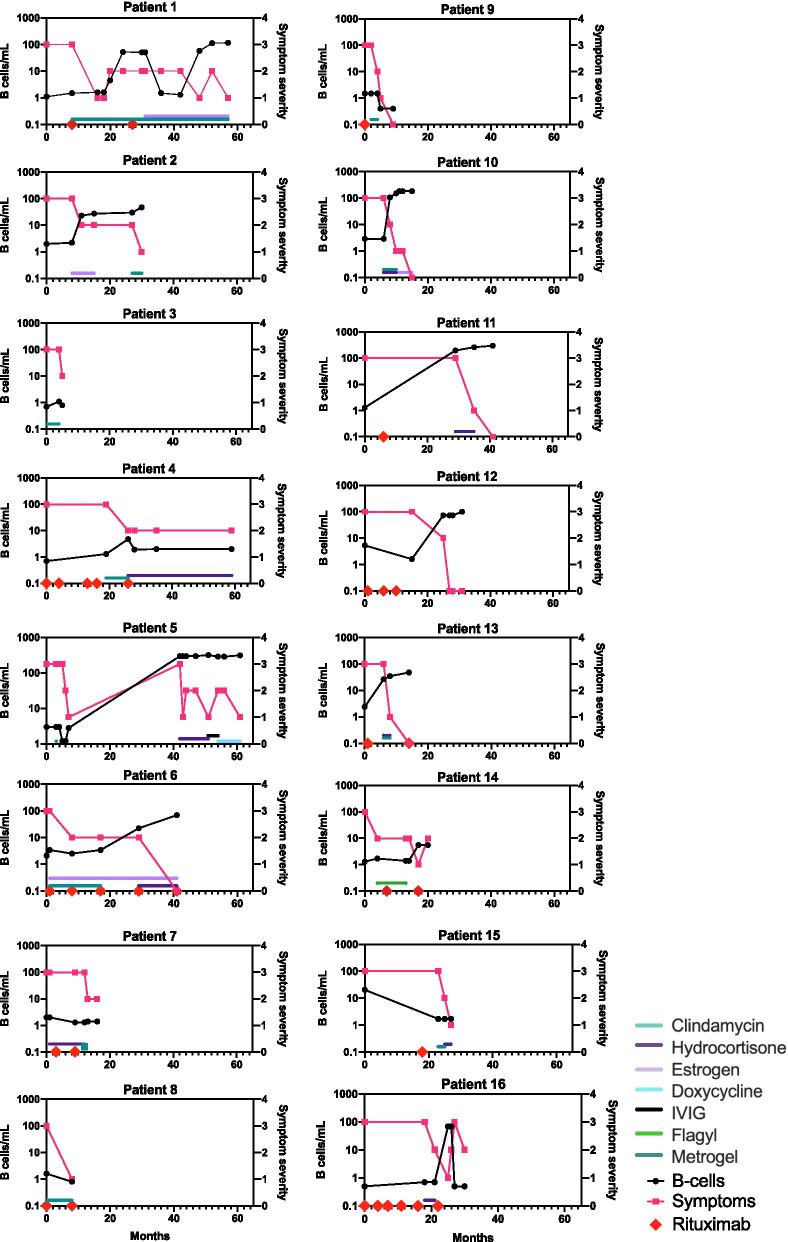
Graph of symptom severity (pink line) and systemic B-cell counts (black line) for the 16 vaginitis cases. Colored lines above the X-axis represent vaginitis-specific treatments, and red diamonds on the X-axis indicate doses of rituximab. Although B-cell counts are not measured immediately after dosing, the expected response is a drop to near zero on the day after infusion. Time zero for each patient is when she recalls the onsent of vaginitis symptoms—which may have been long before reporting those symptoms to providers

Most women (15/16, 94%) were treated with the recommended therapy for desquamative inflammatory vaginitis (DIV): vaginal clindamycin or intravaginal steroids (Fig. 1). Other treatments that patients received include systemic steroids, IVIG, vaginal estrogen, metronidazole, colchicine, boric acid, oral or topical anti-fungals, and oral antibiotics (including doxcycycline, cefuroxime, trimethoprim/sulfamethoxazole, amoxicillin clavulanate, nitrofurantoin, levofloxacin, ciprofloxacin). For 8/16 (50%) women, B cells returned after discontinuation of rituximab. Among women with return of B cells (defined as > 10 cells/mL), symptoms either completely (5/9; 56%) 5/8 or mostly (4/9; 44%) improved. In the remaining seven without B cell return, symptoms mostly (3/7; 43%) or only partially (3/7;43%) improved, with only one person achieving resolution of symptoms. At the time of evaluation for this study, the median duration of vaginitis symptoms was 18 months (range 5 to 61 months) with 10/16 patients continuing to have symptoms.

To assess potential risk factors for development of vaginitis, forty-one controls, matched to vaginitis cases by age, diagnosis, and rituximab-exposure were selected from the study population. Demographic, medical and laboratory characteristics of cases and controls are included in Table 1. Women with inflammatory vaginitis were more likely to report a history of urinary tract infection (UTI): 50% vs 17% (*p* = 0.01). There was no difference in white blood cell (WBC) counts, T cell counts, IgA, IgM, and IgG levels, and creatinine between cases and controls (Table 1). Neutropenia was seen in more cases than controls (25% vs. 12%), more cases received cyclophosphamide (88% vs. 66%, *p* = 0.1) and total cumulative cyclophosphamide dose was higher in cases (*p* = 0.06), though these differences did not reach statistical significance. There were no significant differences in co-morbidities between cases and controls (Table 1).

**Table 1:** Comparison of demographic, medical and laboratory characteristics of cases and controls. Laboratory values are from the date closest to diagnosis of vaginitis (cases), or the matched rituximab dose timing (for controls)

	Cases (N = 16)	Controls (N = 41)	*P* value
Age	44 ± 14	46 ± 17	0.69
*Diagnosis*			0.53
ANCA vasculitis	11 (69%)	33 (80%)	
Lupus nephritis	1 (6%)	3 (7%)	
Other	4 (25%)	5 (13%)	
Rituximab doses	13 ± 7	12 ± 7	0.7
Longest duration of B cell depletion	30 (27, 69)	30 (18, 53)	0.5
Cumulative duration B cell depletion (months with < 5 cells/uL)	47 (29, 84)	38 (19, 68)	0.2
CD3 (cells/uL)	1363 ± 799	1102 ± 550	0.17
CD4 (cells/uL)	863 ± 471	768 ± 333	0.43
CD8 (cells/uL)	428 ± 329	311 ± 204	0.14
WBC (thousand cells/uL)	8.0 ± 2.6	8.1 ± 3.4	0.96
IgG (mg/dL)	777 (593, 948)	692 (600, 843)	0.65
IgM (mg/dL)	26 (21, 63)	43 (22, 79)	0.46
IgA (mg/dL)	129 (61, 156)	128 (88, 173)	0.55
Ever IgG < 600 mg/dL	7 (44%)	19 (48%)	0.74
Ever IgM < 20 mg/dL	4 (25%)	9 (23%)	0.88
Ever IgA < 80 mg/dL	4 (25%)	7 (18%)	0.55
Cyclophosphamide	14 (88%)	27 (66%)	0.10
Cumulative cyclophosphamide dose [15]	750 (400, 1025)	375 (0, 750)	0.06
Late-onset neutropenia	4 (25%)	5 (12%)	0.23
Urinary tract infection	8/16 (50%)	7/41 (17%)	0.01
*Co-morbidities*			
Hypertension	6 (38%)	21 (51%)	0.35
COPD	1 (6%)	1 (2%)	0.48
Asthma	0 (0%)	3 (7%)	0.27
DVT	1 (6%)	3 (7%)	0.89
Colitis/UC/Crohn’s	2 (13%)	1 (2%)	0.13
Diabetes	0 (0%)	3 (7%)	0.27

Values are expressed as N(%), mean ± SD, or median (interquartile range)

*COPD* chronic obstructive pulmonary disease, *DVT* deep vein thrombosis, *UC* ulcerative colitis

## Discussion

This case series highlights inflammatory vaginitis as a potential side effect of prolonged B cell depletion in patients treated with rituximab therapy for autoimmune disorders. Case patients developed symptoms that include vaginal discharge and pain. Most patients with return of B cells after cessation of rituximab had improvement in or resolution of vaginal symptoms however, all patients who have not had B cell recovery have persistent symptoms. Overall, women < 50 years of age were more likely to develop vaginitis than older women. A case control analysis, comparing women diagnosed with vaginitis to age- and diagnosis-matched controls, found that women with vaginitis were more likely to have received cyclophosphamide, and to have reported prior UTIs, but did not have significant differences in systemic immune parameters.

The inflammatory vaginitis we observed in our case patients has some parallels with two other mucocutaneous disorders of the vagina. The first, desquamative inflammatory vaginitis (DIV), is an inflammatory vaginitis of unknown etiology [9–11]. It is rare and poorly understood, defined by presence of vaginal symptoms (vaginal discharge, dyspareunia, vaginal itching, and vaginal discomfort), vaginal inflammation on exam (erythema and friability), vaginal pH > 4.5, and wet mount findings of increased inflammation (> 1 WBC/epithelial cell) and parabasal cells in the absence of a diagnosed vaginal infection [10]. Many of the women in our study meet at least some of the criteria for DIV. However, our participants differed in demographic characteristic compared to previous reports of DIV: the incidence of DIV is higher in patients older than 50 years, whereas we found a higher incidence of rituximab-associated vaginitis in patients below 50 years [12]. A prior analysis of 130 patients with DIV demonstrated significant improvement in symptoms over a median of 3 weeks in 86% of patients treated with vaginal clindamycin or hydrocortisone, which is significantly better than the response to the same treatments seen in patients in this series [11]. The second clinical entity is vulvovaginal pyoderma gangrenosum (PG) which has been described in case reports of women being treated with rituximab [5–8, 13]. The most salient clinical feature of PG is ulcerations of the vulva, vagina, and/or perianal region, whereas only 1 patient in our series had ulcerations. Notably, the majority of patients in these series were receiving rituximab therapy for treatment of malignancies. The unique constellation of features we observe in our series suggests this to be a distinct subtype of inflammatory vaginitis.

A potential mechanism for this rituximab-associated inflammatory vaginitis is unclear. B cells and antibodies have been implicated in controlling the gut microbiome, thus it is conceivable these factors may also be influential for the vaginal microbiome [14]. We hypothesize that B cell depletion could dysregulate the antibody response to pathogenic and commensal microorganisms in the vagina, allowing overgrowth and inflammation. We were not able to identify any immune parameters such as hypogammaglobulinemia or neutropenia which were associated with developing vaginitis. Patients with vaginitis were more likely to have a history of UTIs. It is possible that this implies a shared risk for infection or inflammation of the uroepithelial and vaginal mucosa. Alternatively, women with vaginitis may develop urethral irritation and have WBC on urinalysis due to the high number in vaginal fluid, leading to a misdiagnosis of a UTI.

While all of the patients that we describe are on rituximab therapy, we do not definitively show that rituximab is the causative agent leading to the inflammatory vaginitis. Other medications including cyclophosphamide could also contribute to decreased estrogen levels or long-term immune abnormalities and increase the risk of this inflammatory vaginitis. Notably, none of our patients developed symptoms while actively receiving cyclophosphamide. It is also possible that the inflammatory vaginitis could be a side effect of the underlying autoimmune diseases or other immune abnormalities related to the underlying autoimmune disease. While many patients did improve with B cell return after stopping rituximab, discontinuation of therapy was not performed in a controlled or randomized way, thus other confounding variables could explain the patients’ improvement. Nevertheless, we hope that our findings highlight vaginitis as potential side effect in patients with autoimmune disease or being treated with rituximab which needs additional evaluation and study.

Our study has several strengths and weaknesses. The strengths are that we leverage a large cohort of patients receiving long-term rituximab therapy. We are also able to use this cohort to identify matched controls to better understand predictors of developing vaginitis. However, our participants receive care at one center so our findings may not be generalizable to other treatment regimens and patient populations. Our study is retrospective, and it is probable that we did not capture the prevalence of vulvovaginal symptoms in our population, as even the women described here did not raise the issue with their nephrology team until symptoms had been going on for many months or years. Prospective, systematic evaluation is needed to determine the true prevalence, and potential association with rituximab therapy.

We describe refractory, inflammatory vaginitis as a potential side-effect of long-term B cell depletion with rituximab therapy in a case–control study. Our report has significant implications for care providers and women who are receiving long-term rituximab therapy (Box 1). Women on rituximab therapy should be screened for symptoms of vaginitis including vaginal discharge and vaginal pain. We recommend referring women who develop symptoms to a gynecologist. It will be important to consider this potential side effect when treating women with long-term rituximab and when studying optimal regimens for long-term rituximab therapy.

**Box 1:** Clinical summary of rituximab-associated vaginitis

*Presentation*: Vaginal discharge (often copious), vaginal pain, pain during sex, vaginal or vulvar irritation	
*Differential diagnosis*: Bacterial vaginosis, vulvovaginal candidiasis, vulvar hypersensitivity, vulvodynia, herpes, cervicitis, sexually transmitted infection	
*Workup to consider*: Vaginal pH, wet mount or gram stain of vaginal fluid, yeast culture or Candida PCR, testing for herpes simplex virus, *Trichomonas vaginalis*, *Neisseria gonorrhoeae* and *Chlamydia trachomatis*	
*Findings suggestive of Rituximab-associated vaginitis*: Vaginal pH > 5, Wet mount or gram stain showing > 1 WBC/epithelial cell per high power microscope field, absence of other infectious etiology	
*Potential Treatments*: Consistent with recommendations for desquamative inflammatory vaginitis [11]	
Vaginal clindamycin 2% cream, 5 g daily for 4–6 weeks **OR** Vaginal hydrocortisone 10% compounded cream, daily for 4–6 weeks **OR** Vaginal hydrocortisone compounded 100 g suppository, daily for 4–6 weeks	
If symptoms persist, continue to significantly affect quality of life, and if medically feasible, a trial of discontinuation of Rituximab to allow B cell return may be benficial	

## Declarations

### Availability of data and materials

The datasets generated and/or analysed during the current study are not publicly available due to lack of IRB approval to share these data with researchers outside of the institution. Additional approval could be sought by corresponding author on reasonable request.
